# Does the variable-stiffness colonoscope makes colonoscopy easier? A meta-analysis of the efficacy of the variable stiffness colonoscope compared with the standard adult colonoscope

**DOI:** 10.1186/1471-230X-12-151

**Published:** 2012-10-24

**Authors:** Qin Xie, Bin Chen, Liu Liu, Huatian Gan

**Affiliations:** 1 Department of Gastroenterology, West China Hospital of Sichuan University, Chengdu, Sichuan Province, 610041, China; 2 Department of Geriatric Medicine and Gastroenterology, West China Hospital of Sichuan University, Chengdu, Sichuan Province, 610041, China

**Keywords:** Colonoscope, Variable-stiffness colonoscope, Stiffness, Meta-analysis

## Abstract

**Background:**

The variable-stiffness colonoscope (VSC) appears to have advantages over the standard adult colonoscope (SAC), although data are conflicting. To provide a comprehensive up-to-date review, we conducted a meta-analysis to compare the efficacies of the VSC and SAC.

**Methods:**

Electronic databases, including PubMed, EMBASE, the Cochrane library and the Science Citation Index, were searched to retrieve relevant trials. In addition, meeting abstracts and the reference lists of retrieved articles were reviewed for further relevant studies.

**Results:**

Eight randomized controlled trials (RCTs), enrolling a total of 2033 patients, were included in the meta-analysis. There was no significant heterogeneity among these studies. The cecal intubation rate was higher with the use of VSC (RR = 1.03, 95% CI 1.01 to 1.06, 8 RCTs). The VSC was also associated with fewer position changes made during colonoscopy. Time to cecal intubation was similar with VSC and SAC (WMD −0.54, 95% CI −1.40 to 0.32) but shorter in subgroup analysis with the use of VSC (WMD = −1.36, 95% CI −2.29 to −0.43). Sedation dose used with the two types of instruments showed no evidence of differences either. For all trials, only patients were blinded because of the nature of the interventions.

**Conclusion:**

Use of the VSC significantly improved the cecal intubation rate and reduced ancillary maneuvers made during the procedure. Cecal intubation time was similar for the two colonoscope types over all trials, whereas a shortened time with the use of the adult VSC was seen in subgroup analysis.

## Background

With the increasing availability of colonoscopy, it has become the most common and accurate tool for detecting structural lesions of the lower gastrointestinal tract and for diagnosing colonic diseases, such as colorectal cancer, polyps and inflammatory bowel disease. However, the presence of sharp angulations or looping always increases the difficulty of the procedure and causes patients distinct discomfort. The failure rate to achieve the cecum initially remains significant at up to 2%-10% [1-3]. Although colonoscopic technical proficiency has demonstrated widespread diffusion throughout the developed world, modifications or improvements should to made to ameliorate outcomes.

The variable-stiffness colonoscope (VSC), which can be incorporated into the standard adult and pediatric colonoscope chasses, has a stiffness control ring with dial setting that ranges from 0 to 3. The endoscopist can adjust the relative flexibility of the scope’s insertion tube. VSC is now available in ‘adult’ and ‘pediatric’ sizes. It has been suggested that this type of colonoscope has a theoretical advantage over the standard adult colonoscope (SAC) with its smaller diameter and greater flexibility. In recent years, several studies have been performed to compare VSC with SAC. However, the results have been inconclusive and could not determine whether VSC is superior to SAC or more suitable for routine adult colonoscopy [4-9]. Therefore, we conducted a meta-analysis to compare the efficacy of VSC with SAC.

## Methods

### Literature search

First, electronic databases, including PubMed (1966 to November 2011), EMBASE (1980 to November 2011), the Cochrane Central Register of Controlled Trials (CENTRAL, The Cochrane Library, Issue 4 of 4, Oct 2011), and the Science Citation Index were searched. The search strategy was performed with the following search terms as both free-text terms as well as MeSH terms: colonoscope, colonoscopy, stiff*, stiffness, variable stiffness colonoscopy, pediatric variable stiffness colonoscopy. Second, meeting abstracts and the reference lists of retrieved articles were reviewed for additional relevant studies. No language restriction was imposed.

### Study selection

Randomized controlled trials (RCTs) comparing VSC with SAC were included for analysis. Only the most recent study was included if more than one study was published using the same study population. Open, uncontrolled, observational studies and case reports were excluded from the meta-analysis.

### Data abstraction

All the data were tabulated with standard data abstractions sheets. For each study and each type of intervention, the following characteristics were extracted: study design and conduct, numbers of patients, endoscopist characteristics, instrument features and study outcomes. Study outcomes included cecal intubation rate, cecal intubation time, sedation dose used, abdominal pain score, and ancillary maneuvers during the procedure (manual pressure used and position changes made).

Two investigators (Xie Q, Chen B) independently extracted details of study population, interventions and outcomes. The paper was reviewed if either one of the two investigators thought an abstract was relevant. If there were any discrepancies about information given in the title and abstract, the full article was reviewed for clarification. Differences in opinion were resolved by discussing with the third author (Liu L).

### Assessment of risk of bias in included studies

For the risk of bias assessment, two investigators independently used an assessment form recommended by the Cochrane Handbook. Any disagreements were resolved by a third author until consensus was obtained. We considered the following criteria:

1. Sequence generation: Was the allocation sequence adequately generated?

2. Allocation concealment: Was the allocation adequately concealed?

3. Blinding: Was knowledge of the allocated intervention adequately prevented during the study?

4. Incomplete outcome data: Were incomplete outcome data adequately addressed?

5. Selective outcome reporting: Were reports of the study free of suggestion of selective outcome reporting?

 6.Other sources of bias: Was the study apparently free of other problems that could put it at a high risk of bias?

Each domain was graded as yes (low risk of bias), no (high risk of bias), or unclear (uncertain risk of bias) according to the criteria.

For ranking the strength and quality of the evidence for a given comparison, the GRADE and Summary of Findings tables recommended by the Cochrane Collaboration were used.

### Assessment of reporting biases

For the assessment of publication bias, a funnel plot was conducted if sufficient data were available.

### Statistical analysis

Meta-analyses were conducted for trials comparing VSC with SAC, using the statistical tool Revman 5.1. Dichotomous data were expressed as relative risk (RR) or odds ratio (OR) and continuous outcomes as the weighted mean difference (WMD) with 95% confidence interval (CI). A fixed effects model was used for pooling of data when statistical heterogeneity was not present. If heterogeneity was existed, a random effects model was performed.

Heterogeneity was quantified with Cochran’s Q test and the I^2^ metric, and 95% CI for I^2^ were calculated. I^2^ was in a scale of 0-100%. If there was “considerable heterogeneity”, which is defined by the Cochrane Handbook for Systematic Reviews of Interventions as an I^2^ value between 75% and 100%, the data were not pooled. When I^2^ >50%, suggesting very large heterogeneity between studies, the random effects model was used and a sensitivity analysis was planned to evaluate heterogeneity among studies.

## Results

### Search results

Overall, 32 articles were identified comparing VSC with SAC. After reading abstracts and full-texts, we excluded 24 of these articles [4-27], because they were reviews or not RCTs. Finally, 8 studies met the criteria for inclusion in the review [28-35].

### Trial characteristics

The characteristics of these studies are summarized in Table 1. All these studies were RCTs, containing a total of 2033 participants (1041 male, 992 female), aged from 15 to 89 years. Previous abdominal or pelvic surgery was reported for 13% of the participants in seven trials [28,30-35]. Seven trials listed procedure indications [28,30-35], with screening colonoscopy or polyp surveillance being the main indications. Five trials compared adult VSC with SAC [28-30,34,35], while the other three were performed with pediatric VSCs [31-33].

**Table 1:** The characteristics of included trials comparing the VSC with the SAC

**study, year, country**	**cecal intubation rate**	**cecal intubation time**	**sedation dose**	**Pain score**	**ancillary maneuvers**	**number of patient (n)**	**colonoscope types**	**endoscopists' experience level**
Akira Horiuchi 2004, Japan	PVSC:95% (117/123) SAC:91% (114/125) P=0.075	Mean(SD),min PVSC:6.8(5.2) SAC:7.5(4.8) P=0.082	Mean, Midazolam,mg PVSC:6.5 SAC:7.3 P=0.76	not stated	Position changes made PVSC:0% SAC:5% P<0.0001 Manual pressure used PVSC:66% SAC:69% P=0.55	PVSC, small-caliber PVSC, SAC	374	Experienced
Al-Shurieki SH 2005,USA	PVSC:95.8% (115/120) SAC:96.6% (114/118) P=1.0	Mean(SD),min PVSC:7.8(5.67) SAC:7.9(3.77) P=0.28	Mean, Meperidine,mg PVSC:56(15) SAC:60(15) P=0.06 Midazolam,mg PVSC:2.2(0.79) SAC:2.5(0.78) P=0.02	Median patient experience scale PVSC:1 SAC:83% SAC:1 P=0.6	position change made PVSC:76% P=0.2 manual pressure used PVSC:29% SAC:32% P=0.64	238	PVSC,SAC	Experienced
Brooker JC 2000,UK	VSC:96.5% (55/57) SAC:90.7 (39/43) P=n.s.	Median(range),min VSC:6min32sec(1 min50sec-19min35sec) SAC:10min35sec(3min45sec-22min35sec) P=0.0005	Median(range) Pethidine,mg VSC:25(0–75) SCA:37.5(0–100)SCA:1.5(0–2.5)	Median Pain Score rated by patients VSC:7(0–82) SAC:24(0–85) P=0.0081	not stated	100	VSC,SAC	Experienced
Darlus Sorbi 2001,USA	VSC:100% (25/25) SAC:88% (22/25) P=0.11	Mean ± SEM VSC:10.6 ± 1.6SAC:10.6 ± 1.7 P=0.97	Mean ± SEM Meperidine,mg VSC:68 ± 7 SAC:67 ± 5 P=0.68 Midazolam,mg VSC:4.3 ± 0.6 SAC:4.1 ± 0.3 P=0.84	Mean ± SEM Pain score reported by patients VSC:1.3 ± 0.4 SAC:1.8 ± 0.6 P=0.64	Mean ± SEM positon changes VSC:0.4 ± 0.1 SAC:1.2 ± 0.4 P=0.46 manual pressure used VSC:0.3 ± 0.1 SAC:1.1 ± 0.4 P=0.05	50	VSC,SAC	limited experienced
Ichiro Yoshikawa 2002,Japan	experienced VSC:99% (103/104) SAC:98% (101/103) P=n.s. Limited experience VSC:98% (127/129) SAC:95 (125/131) P=n.s.	experienced VSC:9.8 ± 6.6 SAC:10.6 ± 7.2 P=n.s. Limited experience VSC:15.7 ± 9.7 SAC:18.5 ± 12.1 P<0.05	not stated	Mean(SD) Pain score rated by patients experienced VSC:1.4 ± 1.1 SAC:1.9 ± 1.1 P<0.01 limited experience VSC:1.7 ± 1.0 SAC:2.1 ± 1.2 P<0.01	Manual pressure used experienced VSC:10% SAC:15% P=n.s. Limited experience VSC:35% SAC:45% P=n.s.	467	VSC,SAC	experienced, limited experience
Lee WH 2007,China	VSC:97% (108/111) SAC:93% (102/110)P=0.28	Mean ± SD VSC:12.4 ± 6.8 SAC:13.2 ± 11.7 P=0.55	Mean(SD) Propofol,mg/kg VSC:0.75 ± 0.65 SAC:0.93 ± 0.62 P=0.02	Mean(SD) Pain score rated by patients VSC:4.6(2.7) SAC:5.9(2.5) P=0.589	Position change made VSC:23% SAC:34% P=0.01 Manual pressure used VSC:23% SAC:37% P=0.08	335	VSC,SAC	Experienced
Shumaker DA 2002,USA	PVSC:94.3% (115/122) SAC:89.8% (114/12) P=0.099	mean(SD) PVSC:9.4(6.8) SAC:7.9(4.5) P=0.089	Mean(SD) Meperidine,mg PVSC:73(23) SAC:77(25) P=0.168 Fentanyl,mg PVSC:93(35) SAC:93(26) P=0.039 Midazolam,mg PVSC:2.9(1.0) SAC:3.0(1.0) P=0.081	Mean(SD) Pain score rated by patients PVSC:3.9(3.2) SAC:4.1(3.0) P=0.589	position change PVSC:33% SAC:33% P=0.96 manual pressure used PVSC:58% SAC:42% P=0.024	363	PVSC,SAC,pediatric colonoscope	Experienced
Sola-Vera J 2011, Spain	VSC:92.9% (52/56) SAC:90.7% (49/54) P=0.7		Mean(SD) VSC:6.14(3.5) SAC:7.7(3.8) P=0.035	Mean(SD) Propofol, mg VSC:155.1 ± 83.3 SAC:176.2 ± 91.2 P=0.2 Fentanyl,mg VSC:0.11 ± 0.03 SAC:0.13 ± 0.04 P=0.06 Midazolam, mg VSC:1.3 ± 0.5 SAC:1.1 ± 0.7 P=0.1	Mean(SD) Pain rated by patients VSC:2.4 ± 4.8 SAC:2.3 ± 4.4 P=0.9	Manual pressure used VSC:44.6% SAC:44.4% P=1.0 position change VSC:12.5% SAC:33.3% P=0.012	124	VSC,SAC	Experienced

The instruments used in the trials included VSCs, pediatric VSCs and SACs. The adult VSCs used were: the Olympus XCF-SH140I (distal tip diameter of 13.2 mm, shaft diameter of 12.9 mm, instrument channel of 3.2 mm, working length 133 cm); the Olympus XCF-SH230L (shaft diameter 12.9 mm, instrument channel 3.2 mm, working length 168 cm); the Olympus CF-240AI (shaft diameter 12.0 mm, instrument channel of 3.2 mm, working length 138 cm); the Olympus CF-Q1402 (shaft diameter of 12.0 mm, instrument channel of 3.2 mm, working length 168 cm) and the Olympus CF-H180AI/L (shaft diameter 12.8 mm, working length of 168 cm). The pediatric VSCs used were: the Olympus XPCF-140AL (insertion tube outer diameter 11.3 mm, instrument channel of 3.2 mm, working length 168 cm); the Olympus PCF-Q260AI (insertion tube outer diameter 11 mm, instrument channel of 3.2 mm, working length of 138 cm) and the Olympus PCF-160AL (insertion tube outer diameter of 11.5 mm, instrument channel of 3.2 mm, working length 168 cm).

The level of experience of endoscopists was evaluated either by years of experience (7 to 20 years) or by the number of procedures done (more than 500 procedures). In the retrieved articles, seven trials evaluated VSC procedures with experienced endoscopists; while two studies evaluated VSC procedures among less experienced endoscopists (one study included both experienced and less experienced endoscopists).

### Risk of bias in included studies

Among the eight RCTs included in this meta-analysis, the allocation sequence was generated using a random number generator [28]; computer-generated random number table [34]; and pre-randomized cards [29]. Four of the eight trials reported adequate allocation concealment [29,30,33,35], while in another four trials the allocation concealment was unclear. The eight trials blinded all patients but none of these trials blinded the endoscopists because of the nature of interventions.

The quality of the evidence for the outcomes for the included studies is shown in the Summary of Findings table (Table 2)

**Table 2:** Summary of findings for the main comparison

**The efficacy of variable-stiffness colonoscopes compared with standard adult colonoscopes**						
**Patient or population: patients with performance of colonoscopy**						
**Settings:**						
**Intervention: variable-stiffness colonoscopes**						
**Comparison: standard adult colonoscopes**						
**Outcomes**	Illustrative comparative risks* (95% CI)		**Relative effect (95% CI)**	**No of Participants (studies)**	**Quality of the evidence (GRADE)**	**Comments**
	Assumed risk	Corresponding risk				
	**Standard adult colonoscopes**	**Variable-stiffness colonoscopes**				
**The cecal intubation rate**	**Study population**		**RR 1.03** (1.01 to 1.06)	1683 (9 studies)	⊕ ⊕ ⊕⊝ **moderate**	
	**933 per 1000**	**961 per 1000** (942 to 989)				
	**Moderate**					
	**912 per 1000**	**939 per 1000** (921 to 967)				
**The cecal intubation time**		The mean the cecal intubation time in the intervention groups was		1583 (8 studies)	⊕ ⊕ ⊕⊝ **moderate**	
		**0.54 lower** (1.4 lower to 0.32 higher)				
**Midazolam used**		The mean midazolam used in the intervention groups was		647 (4 studies)	⊕ ⊕ ⊕⊝ **moderate**	
		**0.03 lower** (0.15 lower to 0.08 higher)				
**Manual pressure used**	**Study population**		**RR 0.92** (0.75 to 1.12)	1533 (7 studies)	⊕ ⊕ ⊕⊝ **moderate**	
	**411 per 1000**	**379 per 1000** (309 to 461)				
	**Moderate**					
	**417 per 1000**	**384 per 1000** (313 to 467)				
**Meperidine used**		The mean meperidine used in the intervention groups was		537 (3 studies)	⊕ ⊕ ⊕⊝ **moderate**	
		**1.32 lower** (3.64 lower to 1.01 higher)				
**Position changes made**	**Study population**		**OR 0.65** (0.47 to 0.89)	1066 (5 studies)	⊕ ⊕ ⊕⊝ **moderate**	
	**375 per 1000**	**280 per 1000** (220 to 348)				
	**Moderate**					
	**333 per 1000**	**245 per 1000** (190 to 308)				

The basis for the assumed risk (e.g. the median control group risk across studies) is provided in footnotes. The corresponding risk (and its 95% confidence interval) is based on the assumed risk in the comparison group and the relative effect of the intervention (and its 95% CI).

*CI* Confidence interval, *RR* Risk ratio, *OR* Odds ratio.

GRADE Working Group grades of evidence.

High quality: Further research is very unlikely to change our confidence in the estimate of effect.

Moderate quality: Further research is likely to have an important impact on our confidence in the estimate of effect and may change the estimate.

Low quality: Further research is very likely to have an important impact on our confidence in the estimate of effect and is likely to change the estimate.

Very low quality: We are very uncertain about the estimate.

### Outcomes

#### Cecal intubation rate

**Figure 1:**
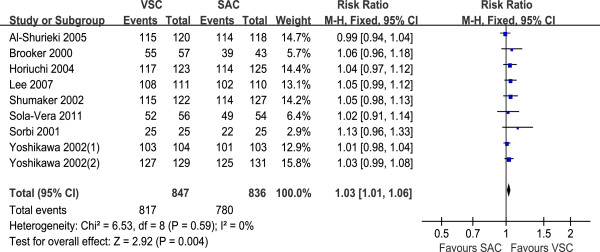
**Cecal intubation rate comparing VSC with SAC; relative risk (RR) with 95% confidence intervals (CI).** Yoshikawa 2002(1) represented the experienced group; Yoshikawa 2002(2) represented the limited experience group. VSC: variable-stiffness colonoscope; SAC: standard adult colonoscope.

Eight trials assessed VSC or pediatric VSC vs. SAC, and the cecal intubation rate was higher in VSC compared with SAC (RR 1.03, 95% CI 1.01 to 1.06, Figure 1). There was no heterogeneity among these studies (I2 = 0%, P = 0.59).

#### Cecal intubation time

**Figure 2:**
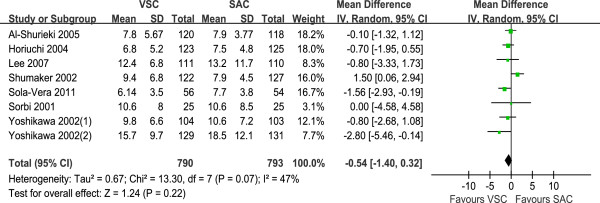
Cecal intubation time comparing VSC with SAC; mean differences with 95% CI.

Meta-analysis of seven trials showed no significant difference of cecal intubation time between SAC and VSC (mean difference −0.54, 95% CI −1.40 to 0.32; Figure 2). There was medium heterogeneity among these seven studies (I2 = 47%, P = 0.07).

#### Sedation dosage

Seven studies reported sedation dose used during the procedure but only five studies listed these data as mean and standard deviation. One trial used a patient-controlled analgesia (PCA) pump consisting of a mixture of propofol and alfentanil [34]; another one employed a combination of midazolam, propofol and fentanyl [35]. Shumaker et al. reported a mixture use of meperidine, midazolam and fentanyl [30], and the last two studies used midazolam and meperidine together [29,33]. Only the doses of meperidine and midazolam intravenously administered were calculated in view of the different types of data recorded. The doses of meperidine (WMD = 1.32, 95%CI −3.64 to 1.01, three trials) and midazolam (WMD = −0.03, 95% CI −0.15 to 0.08, four trials) were similar with the use of either VSC or SAC.

#### Abdominal pain

Five studies presented pain scores as mean and standard deviation. However, the scales used for scoring pain were different. In two studies [30,34], a 0 to 10 score scale was used, and the other three used 0 to 9, 0 to 4 and 0 to 100 visual analogue scales, respectively [29,31,35]. Due to the differences in the scale, we did not pool the data for these studies.

#### Ancillary maneuvers

**Figure 3:**
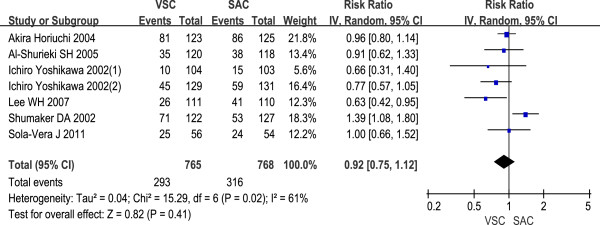
Use of abdominal pressure during colonoscopy with VSC and SAC; relative risk (RR) with 95% CI.

Seven trials listed the data on abdominal pressure use during the procedure. The odds for the use of abdominal pressure during the procedure were similar in both groups (RR 0.92, 95% CI 0.75 to 1.12, Figure 3).

**Figure 4:**
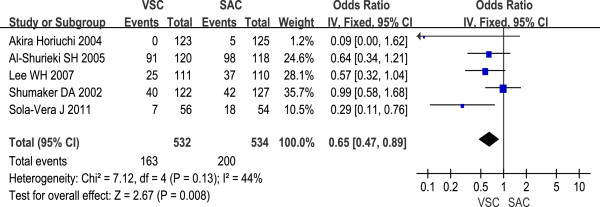
Position changes made during colonoscopy with VSC and SAC; odds ratios (OR) with 95% CI.

VSC was associated with fewer position changes made during colonoscopy (OR 0.65, 95% CI 0.47 to 0.89). The meta-analysis among the five studies showed low heterogeneity (I^2^ = 44%, P = 0.13, Figure 4).

#### Subgroup and sensitivity analysis

To assess the effect of heterogeneity, subgroup and sensitivity analysis were conducted.

**Figure 5:**
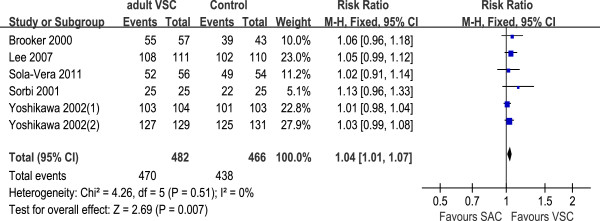
Cecal intubation rate: subgroup analysis of trials comparing adult VSC with SAC; relative risk (RR) with 95% CI.

**Figure 6:**
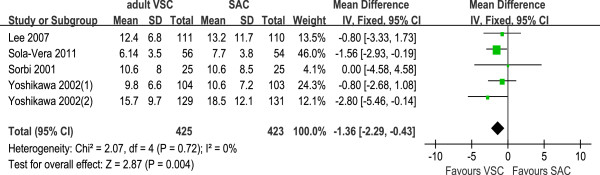
Cecal intubation time: subgroup analysis of trials comparing adult VSC with SAC; mean differences with 95% CI.

Subgroup analysis was done to evaluate the cecal intubation rate and time during colonoscopy, according to the type of VSC (adult or pediatric VSC). The cecal intubation rate with pediatric VSC was similar to that of SAC (OR 1.57, 95% CI 0.85 to 2.90, three trials), while the odds for achieving cecal intubation were a little higher with adult VSC than SAC (RR = 1.04, 95% CI 1.01 TO 1.07, six trials, Figure 5). The cecal intubation time was similar between pediatric VSC and SAC (WMD = 0.19, 95% CI −1.04 to 1.41, three trials.), while comparing adult VSC with SAC, the cecal intubation time was relatively shorter during the procedures for adult VSC (WMD = −1.36, 95% CI −2.29 to −0.43, five trials, Figure 6).

Sensitivity analysis was performed to detect the effect of any one of the included trials on the overall estimate by excluding one of them according to sample size. There were no significant changes to ORs or RRs and WMDs when excluding any one of the included trials.

To detect publication bias, asymmetry was explored in a funnel plot. Distribution of the results of each study in a funnel plot ruled out any potential publication bias.

## Discussion

This meta-analysis included eight RCTs published up to November 2011, including a total of 2033 participants who received VSC or SAC. VSC showed improved cecal intubation rate, as well as decreased position change, but no evidence of advantages in cecal intubation time, sedation dose and manual pressure used. Outcomes were also analyzed in two subgroups based on the type of instruments (pediatric VSC and adult VSC) to evaluate cecal intubation rate and time. By contrast, adult VSC shortened the time to achieve cecal intubation compared with SAC, which was the only difference compared with the results considering all groups together.

In the comparison of VSC with SAC, none of the individual studies had shown an advantage in terms of the frequency of cecal intubation, but the pooled data slightly favored VSC. The increased sample size could be the most significant reason explaining the difference in cecal intubation rates. Large numbers of participants reduced the sampling error, which influenced the significance of the difference of the cecal intubation rates between VSC and SAC. This result is meaningful in clinical practice since, as we know, the failure rate for cecal intubation remains high with the use of SAC, so that this part of the anatomy does not receive clear and early diagnosis and treatment. VSC increased the intubation rate, which contributes to an early and accurate diagnosis.

The individual studies included in this meta-analysis had yielded somewhat conflicting data on cecal intubation time. Five trials found no significant difference in the time to cecal intubation, whereas the other 3 reported significantly shortened time to reach the cecum with VSC. The pooled results with all trials showed no significance; however, subgroup analysis reported shortened time with adult VSC.

Compared with the meta-analysis performed by M. O. Othman et al. [11], our results showed differences in sedation dose. In the subgroup analysis of our meta-analysis, the sedation dose used during colonoscopy was similar between VSC and SAC, but had been reduced with the use of VSC in the previous analysis [11]. In addition, we did not pool the data of pain scores for patients due to the differences in the scale.

Several other individual publications have reported discrepant results with the use of VSC and SAC. For example, Odori et al. [6] reported a prospective RCT of two prototypes of instruments in 352 consecutive cases and found that the cecal intubation time was significantly shorter with the use of VSC. VSC also reduced the need for abdominal pressure and position changes. Rex et al. [8] evaluated the cecal intubation time in a cohort study of 358 consecutive sedated participants amongst VSC, pediatric VSC and SAC. No significant difference was found in the time to reach the cecum. Kaffes et al. [9] found no evidence of difference in cecal intubation time between the two colonoscopic instruments in a nonrandomized trial of 803 participants.

In addition, different methods of activating the variable-stiffness function were used in the included studies and might explain part of the variability. In 2003, Ginsberg [4] described a ‘standard’ technique for using the VSC: the colonoscopy is started with the ‘minimum’ or ‘soft’ control ring (dial setting 0) until looping occurs or the sigmoid colon is traversed. Then, the users reduce the loop, straighten the colonoscope and increase the stiffness control to the ‘hard’ position (dial setting 3). If a loop forms again, the stiffness dial is turned to the ‘soft’ position and the process above is repeated. Horiuchi et al. [32], Sorbi et al. [29] and Sola-Vera J et al. [35] used the same approach described by Ginsberg. Yoshikawa et al. [31] adopted a similar technique to Ginsberg’s, and applied abdominal pressure when activation of the maximum setting (dial setting 3) failed to advance the colonoscope. Al-Shurieki et al. [33] made a slight modification, using the stiffening feature intermittently. When significant looping occurred, dial setting 2 was employed initially,and then stiffening to dial setting 3 was applied if the second setting failed. Lee et al. [34] began at default dial setting 0 and activated the stiffer modes (dial settings 2 or 3) if looping was encountered. Shumaker et al. [30] activated the maximum stiffness mode when the colonoscope was inserted to 30 cm from the anus and left the stiffness setting to full ‘on’ position until the cecum was achieved. The stiffness mechanism was deactivated during withdrawal. In almost all the studies reviewed above, the variable stiffness function was activated when looping was encountered. Furthermore, Shah et al. [7] performed an RCT to evaluate the effect of routinely stiffening the straightened VSC after traversing the sigmoid colon, finding that with the stiffening function activated, the time needed to negotiate the proximal colon and splenic flexure shortened and ancillary maneuvers were reduced. These results may reflect another way in which to use the VSC in clinical practice and may explain the differences in cecal intubation time in comparison with SAC.

There were no scope-related complications reported in the studies included in this meta-analysis. To this point, no safety concerns have been raised with the use of VSCs. However, a single case report draws a possible association between the use of pediatric VSC and a sigmoid perforation, and only the distal descending colon was reached in a patient with a fixed and angulated sigmoid colon [5]. During the procedure, precise judgment and caution must be used, especially when advancing through a narrowed colon or pushing through loops.

A potential limitation of this meta-analysis is that these studies could not be performed to ‘blind’ the endoscopists to the nature of the interventions. Additionally, different models and manufacturers of VSC were used in the studies included. Furthermore, indications for activating the variable stiffness function did not follow the same criteria. There was no universal method for using VSC across the studies and across large tertiary centers, which may limit generalization to other practice settings. Finally, in several studies, specific patient subsets, such as colonic cancer and prior colonic surgery, were excluded.

## Conclusions

In conclusion, the present meta-analysis demonstrated that the VSC was associated with a higher likelihood of achieving cecal intubation and with fewer position changes. However, for most patients, they don’t matter which instrument was used. The more important problem appears to be how to translate these results of colonoscope trials into clinical practice. It might be difficult to predict beforehand which patients will have fixed, angulated sigmoid colons or long, floppy colons. Therefore, there may be no optimal colonoscope model for all patients and endoscopists at all times [4,10] and further studies should be performed to confirm the role of VSC.

## Competing interests

The authors declare that they have no competing interests.

## Author's contributions

QX participated in the design of the study, performed the statistical analysis and drafted the manuscript. BC participated in the statistical analysis and helped to draft the manuscript. LL carried out the statistical analysis. HTG participated in the design of the study. All authors read and approved the final manuscript.

## Pre-publication history

The pre-publication history for this paper can be accessed here:

http://www.biomedcentral.com/1471-230X/12/151/prepub
